# The influence of somatosensory and muscular deficits on postural stabilization: Insights from an instrumented analysis of subjects affected by different types of Charcot–Marie–Tooth disease

**DOI:** 10.1016/j.nmd.2015.05.003

**Published:** 2015-08

**Authors:** Tiziana Lencioni, Giuseppe Piscosquito, Marco Rabuffetti, Gabriele Bovi, Daniela Calabrese, Alessia Aiello, Enrica Di Sipio, Luca Padua, Manuela Diverio, Davide Pareyson, Maurizio Ferrarin

**Affiliations:** a Biomedical Technology Department, IRCCS Foundation Don Gnocchi Onlus, Milan, Italy; b Unit of Clinic of Central and Peripheral Degenerative Neuropathies, IRCCS Foundation, C. Besta Neurological Institute, Milan, Italy; c Department of Neuroscience, Ophthalmology and Genetics, University of Genoa, Genoa, Italy; d Centro S. Maria della Pace, Foundation Don Gnocchi Onlus, Rome, Italy; e Polo Riabilitativo del Levante Ligure, Foundation Don Gnocchi Onlus, Sarzana, Italy

**Keywords:** Charcot–Marie–Tooth disease, Large and small sensory fibers, Muscle weakness, Balance impairment, Rehabilitation

## Abstract

Charcot–Marie–Tooth (CMT) disease is the most common hereditary neuromuscular disorder. CMT1 is primarily demyelinating, CMT2 is primarily axonal, and CMTX1 is characterized by both axonal and demyelinating abnormalities. We investigated the role of somatosensory and muscular deficits on quiet standing and postural stabilization in patients affected by different forms of CMT, comparing their performances with those of healthy subjects. Seventy-six CMT subjects (CMT1A, CMT2 and CMTX1) and 41 healthy controls were evaluated during a sit-to-stand transition and the subsequent quiet upright posture by means of a dynamometric platform. All CMT patients showed altered balance and postural stabilization compared to controls. Multivariate analysis showed that in CMT patients worsening of postural stabilization was related to vibration sense deficit and to dorsi-flexor's weakness, while quiet standing instability was related to the reduction of pinprick sensibility and to plantar-flexor's weakness. Our results show that specific sensory and muscular deficits play different roles in balance impairment of CMT patients, both during postural stabilization and in static posture. An accurate evaluation of residual sensory and muscular functions is therefore necessary to plan for the appropriate balance rehabilitation treatment for each patient, besides the CMT type.

## Highlights

• We studied the role of sensory and muscular deficits in balance impairments in CMT.
• Large sensory fibers and dorsi-flexor muscles affect the dynamic phase of stabilization.
• Small sensory fibers and plantar-flexor muscles influence the static phase of balance.
• Residual sensory and muscle functions must be evaluated for a proper rehabilitation.

## 1 Introduction

Charcot–Marie–Tooth (CMT) disease represents a heterogeneous group of inherited peripheral neuropathies and, with a prevalence of one case in 2500 people [1], is the most common hereditary neuromuscular disorder. It is characterized by symmetrical, slowly progressive muscular weakness and wasting, and sensory impairment, with a length-dependent pattern. Therefore, distal lower limbs are usually more and earlier affected [2]. Despite wide genetic heterogeneity of disease (more than 70 causative genes of CMT and related disorders have been identified thus far), the phenotypes of the different CMT subtypes are relatively similar and usually characterized by foot deformities, loss of deep tendon reflexes, distal muscle weakness and atrophy, decreased touch, pain and vibration sensation [3], that altogether affect walking [4] and balance [5,6]. There are two main forms of CMT: CMT1, characterized by a primary demyelinating process, and CMT2, which is primarily an axonal disorder. The X-linked variety CMTX1, associated with *GJB1* (gap-junction B1) gene mutations, has a mixed pattern [2,7,8] by showing both myelin and axonal abnormalities and decreased nerve conduction velocities (NCVs) often in the range intermediate between CMT1 and CMT2. The CMT1A subtype, associated with the *PMP22* (peripheral myelin protein 22) gene duplication, is the most common CMT form accounting for approximately 45–50% of all CMT cases, while CMT2 and CMTX1 are less frequent, accounting for about 17–25% and 8–10% of all CMT cases, respectively [1,9]. Currently, no effective pharmacological therapy is available and physiotherapy remains the only suggested treatment [10,11]. Although there is no established CMT-specific rehabilitation program, patients are often treated with tendon stretching exercises, muscle strengthening, balance and gait training. Balance impairment is one of the major problems in CMT patients, resulting in further standing and gait difficulties, increased fall risk and reduced quality of life [12,13]. There is still uncertainty, however, about which muscles and sensory fibers are responsible for such imbalance.

The following somatosensory fibers are affected in CMT patients: large (Aα) and medium sized myelinated (Aβ), small myelinated (Aδ), and unmyelinated (C). Large and medium sized nerve fibers carry motor function, sense of joint movement and position, vibration sense and discriminative and fine touch sensation. Small fibers convey temperature, pain sensation and pressure touch sensation as well as autonomic functions [14,15]. Loss of vibration sense is mainly due to the damage to large fibers [14,16] that are characterized by very rapid adaptation, whereas decrease of pinprick sensation is related to loss of smaller myelinated and unmyelinated fibers, which have slow adaptation [14,17]. In CMT1A, characterized by myelin sheath abnormalities, the large myelinated fibers are typically more affected than the smaller myelinated or unmyelinated fibers. CMT2 is usually characterized by a more widespread process where primary axonal degeneration usually affects all caliber fibers [15,18,19].

Peripheral neuropathies such as CMT affect motor control, which relies, among other factors, also on somatosensory system integrity. Therefore, peripheral nerve damage may affect postural and/or motor performances by altering the afferent information flow. Although there is evidence that after a rehabilitation treatment CMT patients' stance became more steady and harmonic [6], there are very few studies on balance in CMT subjects. Most of the works focused on the characterization of walking, where the main deficits affecting the patients are the foot drop and the push-off failure [4,20,21], or on longitudinal studies to monitor disease progression [22,23].

Nardone et al. [18,24] hypothesized that neuropathy affects stance balance when smaller fibers are compromised in addition to large ones. The authors reached this conclusion as they did not find differences in stance balance when comparing healthy subjects with mildly affected CMT1A subjects, who are likely to have only slight impairment of small fibers. CMT2 subjects, suffering from the axonal form, behaved differently and were unstable compared to both CMT1A and healthy subjects.

A recent study pointed out that the somatosensory system is not sufficient to maintain balance: Lencioni et al. [5] found that CMT1A subjects may also have balance difficulties and may be less stable than controls during quiet stance. Greater difficulty to maintain erect posture was mainly associated with plantar-flexor muscle weakness, rather than to somatosensory system damage, a reasonable finding since plantar-flexor muscles are considered important in maintaining the standing posture [25]. Other studies supported these results. Rossor et al. made the clinical observation that the ankle plantar-flexor's weakening in CMT patients has major functional consequences on postural skills [26]. Guillebastre et al. found in a group of CMT patients a significant correlation between plantar-flexor muscular deficit and worsening of postural parameters, evaluated through a dynamometric platform [27].

To the best of our knowledge, no study has investigated the postural ability of CMTX1 subjects thus far, although this is the second most common CMT type, and overall few data are available on balance control of subjects with different types of CMT. The role of somatosensory and muscular systems on postural stability of these patients deserves further investigation. Therefore, the goal of the present study was to clarify the distinctive contributions of (a) distal muscular weakness and (b) loss of different somatosensory afferents on balance control in CMT, both during static and dynamic conditions. To this aim, we enrolled a large number of CMT subjects, showing genetic heterogeneity and a wide range of severity of somatosensory fibers and muscle strength affection.

As an assessment method, we chose that proposed by Rabuffetti et al. [28], which is based on dynamometric platform data to evaluate the postural stabilization occurring during transition from sitting to erect posture [28]. Very recently, this method has been successfully applied in studies regarding postural stabilization and balance assessment in people with multiple sclerosis [29] and CMT1A [5]. An interesting aspect of this approach is that it explores both a dynamic action (the postural stabilization) and a static condition (the quiet erect posture). In comparison with classical static posturography, such assessment provides a more extensive and global information about motor control abilities of the examined subjects.

## 2 Materials and methods

### 2.1 Subjects and clinical evaluation

Seventy-six Charcot–Marie–Tooth adult subjects (40 females, age: 43.1 ± 13.9 years range [18–70], height: 167.3 ± 11.1 cm, weight: 66.2 ± 14.8 kg) affected by CMT1A (47 subjects, 29 females, age: 44.5 ± 12.0 years, height: 166.2 ± 11.2 cm, weight: 67.0 ± 14.8 kg), CMT2 (13 subjects, 9 females, age: 48.9 ± 15.8 years, height: 162.7 ± 9.3 cm, weight: 57.2 ± 13.5 kg) and CMTX1 (16 subjects, 2 females, age: 34.2 ± 14.0 years, height: 169.4 ± 10.9 cm, weight: 67.2 ± 14.1 kg), with a wide range of ages and disease severity, were enrolled for the present study. Among CMT2 subjects, three had mutations in the Myelin Protein Zero gene (*MPZ*) and seven in the Mitofusin 2 gene (*MFN2*), while in the remaining three no mutation has been identified so far. Forty-one healthy subjects, without any walking and sensory impairments, formed the control group which was comparable to the whole group of CMT subjects for age and sex (21 females, age: 44.2 ± 19.0 years range [18–72], height: 169.0 ± 10.7 cm, weight: 68.2 ± 14.5 kg). No patient presented foot deformities so relevant to affect their postural performance.

According to the Charcot–Marie–Tooth Examination Score (CMTES; ranging from 0, normal, to 28, worst [30]), the following items were quantified: pinprick sensation, vibration sense, legs strength, arms strength, sensory symptoms, motor symptoms – legs and motor symptoms – arms. Ankle plantar-flexor and dorsi-flexor muscles' strength was assessed by means of the Medical Research Council scale (*MRC_APF_* and *MRC_ADF_*; 0 indicating no force, 5 full strength [31]).

### 2.2 Protocol and instrumental analysis

A sit-to-stand (STS) task was performed as previously described [28]: the subject, in an opened eyes condition and keeping his/her feet still on a force platform embedded in the floor, was asked to move from the initial sitting position to a standing one, without the help of hands, and to stand as still as possible for at least 20 s in an upright posture, looking at a target placed at eye level 1 m away. The STS task was repeated three times. The dynamics associated to the performance of the STS task was evaluated by means of the ground reaction force (GRF) measured by a piezoelectric force platform (Kistler, Switzerland, 960 Hz). Acquired data were analyzed as already described [28]. Briefly, for each STS trial, the root mean square of the antero-posterior component (RMS_AP_) of the GRF was computed in 1 s moving window. After synchronization of the three RMS_AP_ profiles to the t_0_ instant, corresponding to the end of macroscopic movement, the median value for each time instant was then computed, providing a median profile. Finally, a decaying function (negative exponential function) was fitted on the median profile, allowing the identification of three independent parameters named *Y*_0_ [m s^−2^], *T* [s] and *Y_inf_* [m s^−2^]. Specifically, *Y*_0_ quantifies the initial instability rate at *t*_0_, *T* is the time duration related to the stabilization needed to reduce instability from *Y*_0_ down to *Y_inf_*, and *Y_inf_* is the final asymptotic instability rate which accounts for the residual long-term postural oscillations. Hereinafter, what happens during the time period between t_0_ and t_0_ + 3T is defined as *postural stabilization phase*, while what happens after *t*_0_ + 3*T* is defined as *quiet standing*. The parameter *I*, defined as *Y*_0_*T [m s^−1^], is a compound index quantifying the stabilization skills of the subjects.

The protocol was approved by the Local Ethics Committee and all subjects provided informed consent before entering the study.

### 2.3 Statistical analysis

All statistical analyses were conducted in MedCalc^®^ for Windows, version 11.5 (MedCalc Software, Ostend, Belgium). The data were not normally distributed, as showed by the Shapiro–Wilk test. Therefore, the Mann–Whitney U test was used to analyze the differences between control and CMT groups.

To evaluate the influence of sensory (pinprick and vibration sense) and muscular (MRC_APF_ and MRC_ADF_) factors on behavioral indices of global postural performance (*I* and *Y*_inf_), the multiple correlation analysis was used. Since *I* and *Y*_inf_ parameters were not normally distributed, a logarithmic transformation was made.

In all analyses, p-values < 0.05 were considered statistically significant.

## 3 Results

All subjects enrolled in the study were able to perform the STS task without using hand support. We found no significant difference in age (p = 0.11), height (p = 0.28) and weight (p = 0.53) among CMT subgroups and the control group. Relevant clinical data of CMT subjects (median and interquartile range) are reported in Table 1. Overall, neurological examination did not reveal any difference based on the type of CMT (CMTES Median (interquartile range): 8 (6–9) for CMT1A, 10 (9–13) for CMT2, 8 (6–10) for CMTX1, p > 0.05). The characteristics of predictive factors among CMT subgroups are reported in Supplementary Fig. S1.

CMT subjects had significantly higher values for all parameters (*T*, *Y_inf_*, *Y*_0_, *I*), meaning worse balance performances, with respect to controls. Such a comparison (median and interquartile range) is reported in Table 2. Even when examining CMT subjects according to their CMT type (CMT1A, CMT2, CMTX1), we found significantly higher parameters *Y_inf_* and *I*, indicative of impaired balance skill of each subgroup as compared to healthy subjects (Supplementary Table S1).

The predictor variables (pinprick sensation, vibration sense, MRC_ADF_ and MRC_APF_) were significantly associated with both global indices, *I* and *Y_inf_* (p = 0.001, F-ratio = 5.39, R^2^-adjusted = 0.23 and p < 0.001, F-ratio = 7.01, R^2^-adjusted = 0.30, respectively). In detail, the parameter *I* was significantly correlated with dorsi-flexor's strength (p < 0.01) and vibration sense (p < 0.05), whereas the *Y_inf_* parameter was significantly related to the strength of plantar-flexors (p < 0.01) and pinprick sensation (p < 0.05) (Table 3).

**Fig. 1:**
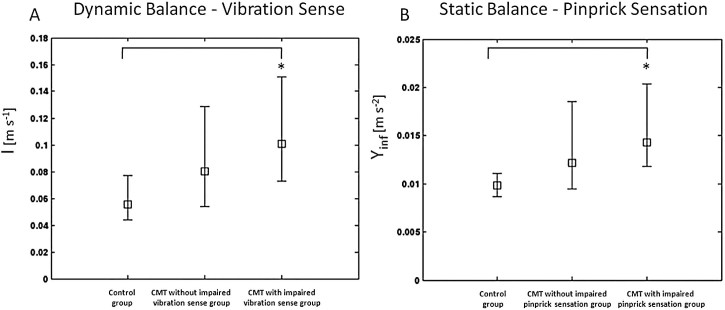
(A) Median and interquartile range values of the parameter for the dynamic balance (*I*) for controls, CMT subjects without impaired vibration sense and CMT subjects with impaired vibration sense. (B) Median and interquartile range values of the parameter for the static (*Y_inf_*) for controls, CMT subjects without impaired pinprick sensation and CMT subjects with impaired pinprick sensation. * indicates a significant difference among groups (p < 0.05) tested by Kruskal–Wallis ANOVA test. *I*: global index of performance during stabilization; *Y_inf_*: the residual instability after stabilization in quiet standing.

To clarify the role of sensory input on balance control, the dynamic balance (*I*) of the CMT subjects, whose vibration sense was impaired (i.e. subjects with CMTES item vibration sense > 0, representing 75% of the sample), was compared with the CMT subjects without vibration sense impairment (i.e. subjects with CMTES item vibration sense = 0, representing 25% of the sample) and that of the healthy subjects. The impaired vibration sense CMT group was less stable compared to the healthy group (p < 0.05) (Fig. 1A). A similar comparison was made for the static balance (*Y_inf_*) dividing the subjects on the basis of the pinprick sense score. The static balance (*Y_inf_*) of the CMT subjects, whose pinprick sensation was impaired (i.e. CMTES item pinprick sensation > 0, accounting for 83% of the sample), was compared with the CMT subjects without pinprick sensation impairment (i.e. CMTES item pinprick sensation = 0, accounting for 17% of the subjects) and that of the healthy subjects. The impaired pinprick sensation CMT group was less stable compared to the healthy group (p < 0.05) (Fig. 1B).

## 4 Discussion

It is known from clinical experience and data that CMT patients show locomotor and/or postural disturbances depending on the progression of the degenerative processes affecting the peripheral nerves. What is still unclear is the different roles played by muscular weakness and somatosensory deficit on postural instability, since CMT patients are usually affected by both dysfunctions. The problem is even more complex because the possible different degrees of impairment of large and smaller sensory fibers may depend on the CMT type. With the aim of clarifying the above picture, in the present study we evaluated the STS task and the subsequent postural stabilization process in a group of patients with different types of CMT and with a wide spectrum of disease severity.

Based on the previously published results, reported in the Introduction, we hypothesized a specific interaction between muscle strength and sensory factors, on the one hand, and two global indices (*Y_inf_* and *I*) of postural stabilization performance on the other hand. These indices evaluate two different balance conditions, static and dynamic, respectively. In fact, *I* is a measure of the balance performance during the stabilization phase just after the global movement of STS, while *Y_inf_* is an index of the residual balance instability in static conditions, once the still standing posture is achieved [28]. In the present study, we have expanded the sample size as compared to the work by Lencioni et al. [5], because it is known that there are differences in the degree of impairment of sensory fibers based on the CMT type; therefore, we recruited CMT subjects other than CMT1A, namely affected by CMT type 2 and CMTX1.

Neither the whole group of CMT patients nor any CMT subgroup was characterized by a normal static (*Y_inf_*) and/or dynamic (*I*) postural control and, since there were no significant differences in both parameters among CMT subgroups, we focus the discussion on the causes of balance impairments on the group as a whole regardless of the CMT types. Our study confirms the difficulties of CMT subjects to control balance during both dynamic and static phases, and elucidates the mechanisms underlying these balance impairments.

The analysis of the behavioral index *Y_inf_*, expression of postural control during quiet standing, confirmed that both sensory and muscular functions are involved in balance performance. In particular, the significant correlation between *Y_inf_* and pinprick sensation, shown by multivariate analysis, highlighted the major role of small sensory fibers during quiet standing. Loss of small fibers has a direct relationship with impaired balance [32], in particular by altering the feedback of the slow adaptive movements [33,34], which are small position adjustments needed for maintenance of static upright posture. Not surprisingly, the reduction of pinprick sensation was found to be associated with an increase in the amplitude of steady state postural sway, a functional gauge of stance instability, as already proposed by Nardone et al. [18]. This is further confirmed by the data shown in Fig. 1B: CMT subjects with pinprick sensation impairment were less stable than controls, whereas stability of CMT subjects without pinprick sensation impairment did not significantly differ from the controls. CMT subjects without pinprick sensation impairment had *Y_inf_* values not significantly different from those of CMT subjects with impaired pinprick sensation; however a clear trend is identifiable from Fig. 1B. This discrepancy could be due to the fact that CMT subjects with relative preservation of small fibers could have balance difficulties related to other factors (e.g. plantar-flexor muscles, as highlighted in the discussion below).

The difficulties in reaching the static balance were also related to plantar-flexor muscles' strength in the light of the correlation found between *Y_inf_* and MRC_APF_, and in line with previous studies [5,25,26].

In a similar way, we also found that the parameter *I*, expression of the subject's ability to stabilize the body during dynamic phases, depends on the functionality of both sensory and muscular systems, although through different factors. In fact, multivariate analysis showed that parameter *I* correlated significantly with the variable MRC_ADF_ and the vibration sense, the former a measure of strength of dorsi-flexor muscles and the latter of functionality of large sensory fibers, more involved during rapid movements than during quiet stance. This result is in accordance with Nardone et al., who suggested that these fibers are involved during dynamic conditions and not during quiet standing [18]. A further confirmation of this finding is derived from data shown in Fig. 1A: CMT subjects with vibration sense impairment were less stable than controls, whereas CMT subjects without vibration sense impairment were as stable as controls. Therefore we can draw the same conclusions that we reached for static balance, about the relationship between vibration sense and the parameter *I* during postural stabilization: CMT subjects with less compromised large fibers stabilize themselves with less difficulty. The role of dorsi-flexor muscles during the stabilization phase of a STS task is an interesting finding of this study: it can be interpreted, in accordance with Goulart et al. [35], as the hallmark of this muscle group as a controller of the postural stabilization in contrast to the propulsive function provided by extensor muscles of the ankle and, even more, of knee and hip joints.

We are aware that the values of correlation coefficient R obtained from the multivariate analysis, despite the statistically significant level attained, correspond to a moderate correlation among factors. This could indicate, as already speculated by other authors, that other systems, such as visual or vestibular system, may play an important role in the balance and postural stabilization [36]. Another explanation of the moderate correlation coefficients observed could be related to the fact that the clinical rate of pinprick, vibration sensibility and muscle strength are ordinal with only a few levels, while the parameters *Y_inf_* and *I* are continuous variables.

The results of the present study highlight the different relationships between postural behaviors and the sensory and muscle systems, depending on the specific balance condition (static or dynamic). These differences are due to the different involvement of sensory fibers: smaller sensory fibers play a crucial role in postural control during quiet standing while the large sensory fibers are involved in the dynamic phase during postural stabilization. On the other hand, the present study confirms the relationship between the muscular deficit and the functional capability both during postural stabilization (involving especially dorsi-flexor muscles) and quiet standing (involving especially plantar-flexor muscles) as we already showed in a previous work [5].

Rehabilitation has long been proposed in CMT, including muscle strengthening, balance training and endurance work with variable times and modalities, but until now, no study has focused on the specific sensory motor deficit. The results of this work suggest that the dynamometric platform may be useful to detect different patterns of imbalance in CMT patients, giving extensive information about the dynamic and static instability and postural stabilization. First, dynamic and static balance may be easily improved by strengthening of the *anterior tibialis* and *triceps surae*, respectively. Second, specific balance training aimed at sensory reinforcement can be better oriented when balance problems are referred by the patients. Proprioception rehabilitation may be suggested when STS difficulties are found, and on the other hand, static balance training proposed when stand imbalance is observed. Furthermore, a compensation may be provided by visual and vestibular afferents, as all these sensory inputs play an important role in maintaining static and dynamic balance. Particularly, strategies aimed at improving compensatory sensory mechanisms are indicated in CMT patients because those sensory inputs are usually spared.

In conclusion, specific sensory and muscular deficits play different roles in balance impairment of CMT patients: our data provided evidence that small sensory fibers and plantar-flexor muscles contribute mainly in the static posture phase, while large sensory fibers and dorsi-flexor muscles are primarily involved during postural stabilization. As a consequence, we believe that a specific evaluation of leg muscles' strength and of the different components of somatic sensation is needed to tailor rehabilitation treatments and to provide hints for a safe balance and locomotion in CMT patients.

## Figures and Tables

**Table 1:** Clinical data of the whole CMT group: median (interquartile range) and the range of values (min–max).

Parameters		Median (interquartile range)	Values range [min–max]
CMTES	Total	8 (6–10)	[0–18]
	Sensory symptoms	1 (0–1.25)	[0–4]
	Motor symptoms – Legs	1 (1–1)	[0–3]
	Motor symptoms – Arms	1 (0–1)	[0–2]
	Pinprick sensation	1 (1–2)	[0–4]
	Vibration sense	1 (0.75–2)	[0–3]
	Strength legs	1.5 (1–2)	[0–4]
	Strength arms	1 (1–2)	[0–3]
MRC	ADF	3.4 (2–4.5)	[0–5]
	APF	5 (4–5)	[0–5]

ADF: ankle dorsi-flexors; APF: ankle plantar-flexors; CMTES: Charcot–Marie–Tooth Examination Score; MRC: Medical Research Council scale for muscle strength.

**Table 2:** Values (median and interquartile range) of postural stabilization parameters for control and CMT groups. Values marked with “*” show significant differences (p < 0.05) between controls and the CMT patients in accordance with U-test.

Parameters	Control group Median (interquartile range)	CMT group Median (interquartile range)
*T* [s]	0.72 (0.47–1.03)	1.13 (0.81–1.59)*
*Y*_0_ [m s^−2^]	0.076 (0.053–0.108)	0.095 (0.073–0.127)*
*Y_inf_* [m s^−2^]	0.010 (0.009–0.011)	0.015 (0.012–0.020)*
*I* [m s^−1^]	0.055 (0.044–0.077)	0.100 (0.072–0.153)*

*I*: global index of performance during stabilization; *T*: time duration of postural stabilization; *Y*_0_: residual instability at the beginning of the stabilization phase; *Y_inf_*: the residual instability after stabilization in quiet standing.

**Table 3:** Correlation coefficients (R) between the dependent variables (*Y_inf_* and *I*) and all the independent variables (pinprick sensation, vibration sense, MRC_ADF_ and MRC_APF_).

Parameters		R-values
Pinprick sensation	*Y_inf_*	0.37*
	*I*	0.14
Vibration sense	*Y_inf_*	0.33
	*I*	0.32*
MRC_ADF_	*Y_inf_*	−0.32
	*I*	−0.42**
MRC_APF_	*Y_inf_*	−0.40**
	*I*	−0.20

The level of statistical significance of R coefficients is indicated with “*” for p < 0.05 and with “**” for p < 0.01.

ADF: ankle dorsi-flexors; APF: ankle plantar-flexors; *I*: global index of performance during stabilization; MRC: Medical Research Council scale for muscle strength; *Y_inf_*: the residual instability after stabilization in quiet standing.

## Appendix Supplementary material

Fig. S1 Sensory and muscular deficits according to CMT type (median and interquartile range). * indicates a significant difference among CMT subgroups (p < 0.05) tested by Kruskal–Wallis ANOVA test. ADF: ankle dorsi-flexors; APF: ankle plantar-flexors; MRC: Medical Research Council scale for muscle strength.

Table S1 Values (median and interquartile range) of postural stabilization parameters for each CMT subgroups according to patients CMT type and control group. Differences among multiple groups (controls, CMT1A, CMT2 and CMTX1) were tested by Kruskal–Wallis ANOVA test. Differences among control and CMT subgroups are marked with “a”, with respect to healthy subjects, and “b”, with respect to CMT1A subjects. *I*: global index of performance during stabilization; *T*: time duration of postural stabilization; *Y*_0_: residual instability at the beginning of the stabilization phase; *Y_inf_*: the residual instability after stabilization in quiet standing.
