# Supplementary material for: The influence of somatosensory and muscular deficits on postural stabilization: Insights from an instrumented analysis of subjects affected by different types of Charcot–Marie–Tooth disease
Source: Neuromuscul Disord. 2015 Aug;25(8):640–5. doi: 10.1016/j.nmd.2015.05.003 (PMC4553554; doi:10.1016/j.nmd.2015.05.003)
Supplement: Fig. S1 — Sensory and muscular deficits according to CMT type (median and interquartile range). * indicates a significant difference among CMT subgroups (p < 0.05) tested by Kruskal–Wallis ANOVA test. ADF: ankle dorsi-flexors; APF: ankle plantar-flexors; MRC: Medical Research Council scale for muscle strength. [file mmc1.zip › Appendix A Figure A.1 Caption.docx.docx]

Appendix A.

Figure A.1: Sensory and muscular deficits according to CMT type (median and interquartile range).

* indicates a significant difference among CMT subgroups (p<0.05) tested by Kruskal-Wallis ANOVA test.

ADF: Ankle Dorsi-Flexors APF: Ankle Plantar-Flexors; MRC: Medical Research Council scale for muscle strength.
