# Supplementary figures and images for: The influence of somatosensory and muscular deficits on postural stabilization: Insights from an instrumented analysis of subjects affected by different types of Charcot–Marie–Tooth disease
Source: Neuromuscul Disord. 2015 Aug;25(8):640–5. doi: 10.1016/j.nmd.2015.05.003 (PMC4553554; doi:10.1016/j.nmd.2015.05.003)

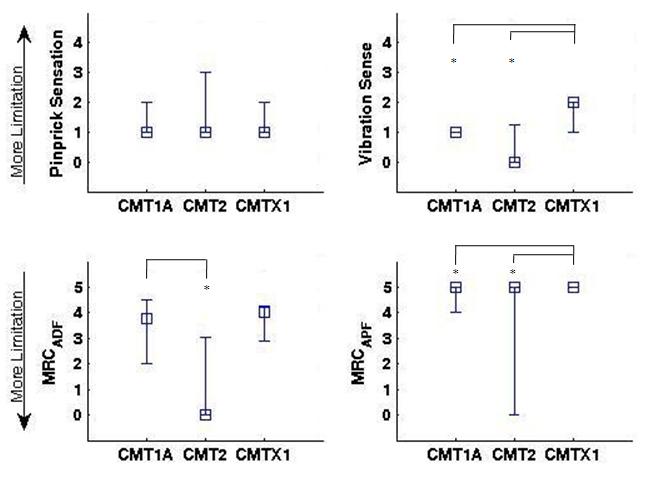

Supplement: Fig. S1 — Sensory and muscular deficits according to CMT type (median and interquartile range). * indicates a significant difference among CMT subgroups (p < 0.05) tested by Kruskal–Wallis ANOVA test. ADF: ankle dorsi-flexors; APF: ankle plantar-flexors; MRC: Medical Research Council scale for muscle strength. [file mmc1.zip › Appendix A Figure A.1 .JPG]
