# Supplementary material for: The influence of somatosensory and muscular deficits on postural stabilization: Insights from an instrumented analysis of subjects affected by different types of Charcot–Marie–Tooth disease
Source: Neuromuscul Disord. 2015 Aug;25(8):640–5. doi: 10.1016/j.nmd.2015.05.003 (PMC4553554; doi:10.1016/j.nmd.2015.05.003)
Supplement: Table S1 — Values (median and interquartile range) of postural stabilization parameters for each CMT subgroups according to patients CMT type and control group. Differences among multiple groups (controls, CMT1A, CMT2 and CMTX1) were tested by Kruskal–Wallis ANOVA test. Differences among control and CMT subgroups are marked with “a”, with respect to healthy subjects, and “b”, with respect to CMT1A subjects. I: global index of performance during stabilization; T: time duration of postural stabilization; Y0: residual instability at the beginning of the stabilization phase; Yinf: the residual instability after stabilization in quiet standing. [file mmc2.docx]

Appendix A.

Table A.1: Values (median and interquartile range) of postural stabilization parameters for each CMT subgroups according to patients CMT type and control group. Differences among multiple groups (Controls, CMT1A, CMT2 and CMTX1) were tested by Kruskal-Wallis ANOVA test. Differences among control and CMT subgroups are marked with “a”, with respect to healthy subjects, and “b”, with respect to CMT1A subjects.

| **Parameters** | | **Control Group**  Median (interquartile range) | | **CMT1A Subgroup**  Median (interquartile range) | | **CMT2 Subgroup**  Median (interquartile range) | | **CMTX1 Subgroup**  Median (interquartile range) | |
| --- | --- | --- | --- | --- | --- | --- | --- | --- | --- |
| **T** | **[s]** | 0.72 | (0.47-1.03) | 1.13 | (0.74-1.60)^a^ | 1.04 | (0.85-1.21) | 1.17 | (0.85-1.47)^a^ |
| **Y_0_** | **[ms^-2^]** | 0.076 | (0.053-0.108) | 0.083 | (0.063-0.102) | 0.135 | (0.085-0.152)^a^ | 0.119 | (0.100-0.146)^ab^ |
| **Y_inf_** | **[ms^-2^]** | 0.010 | (0.009-0.011) | 0.014 | (0.011-0.019)^a^ | 0.018 | (0.015-0.028)^a^ | 0.013 | (0.013-0.020)^a^ |
| **I** | **[ms^-1^]** | 0.055 | (0.044-0.077) | 0.089 | (0.067-0.120)^a^ | 0.156 | (0.084-0.161)^a^ | 0.118 | (0.092-0.160) ^a^ |

I: global index of performance during stabilization; T: time duration of postural stabilization; Y_0_: residual instability at the beginning of the stabilization phase; Y_inf_: the residual instability after stabilization in quiet standing.
